# Association between Fok I vitamin D receptor gene (VDR) polymorphism and impulsivity in alcohol-dependent patients

**DOI:** 10.1007/s11033-014-3607-6

**Published:** 2014-07-25

**Authors:** Małgorzata Wrzosek, Andrzej Jakubczyk, Michał Wrzosek, Beata Kaleta, Jacek Łukaszkiewicz, Halina Matsumoto, Kirk Brower, Grażyna Nowicka, Marcin Wojnar

**Affiliations:** 1 Department of Pharmacogenomics, Medical University of Warsaw, Banacha 1, 02-097 Warsaw, Poland; 2 Department of Psychiatry, Medical University of Warsaw, Warsaw, Poland; 3 Department of Internal Medicine and Diabetology, Medical University of Warsaw, Warsaw, Poland; 4 Department of Biochemistry and Clinical Chemistry, Medical University of Warsaw, Warsaw, Poland; 5 Department of Psychiatry, University of Michigan, Ann Arbor, MI USA

**Keywords:** Alcohol dependence, Impulsivity, Vitamin D receptor gene, Genetic polymorphism

## Abstract

Vitamin D appears to have an important role in the modulation of the central nervous system. Vitamin D exerts its biological effects through its interaction with the vitamin D receptor (VDR). Located on chromosome 12 (12q13.1), the VDR gene has many different polymorphisms. Some of them are known to affect the VDR function, such as FokI (rs2228570, T/C) single nucleotide polymorphism. We aimed to explore a potential relationship between FokI *VDR* polymorphism and impulsiveness in alcohol-dependent (AD) patients. The study population consisted of 148 patients diagnosed with alcohol dependence (DSM-IV criteria) and 212 healthy controls. DNA was extracted from whole blood samples using the standard procedure. Genotypes were analyzed using a real-time PCR method. We found that FokI *VDR* gene polymorphism was associated with impulsivity [Barratt Impulsiveness Scale (BIS)-11 total score; *P* = 0.014], and with attentional impulsivity (BIS-11 subscale; *P* = 0.002) in the male AD patients. Our results suggest that CC FokI genotype of the *VDR* gene is associated with a higher level of impulsivity in these patients. This finding supports the hypothesis that impulsiveness, which significantly contributes to development of alcohol dependence, has a genetic background.

## Introduction

Alcohol dependence may be defined as loss of control over alcohol consumption, which is continued despite the harmful consequences that follow. Alcoholics are not able to drink alcohol in a controlled manner, even if it threatens their health, work or family. Certain personality features are likely to contribute to the development of alcohol dependence. These can include, for example, high levels of impulsivity or poor impulse control, which are considered both a predictor of the development of alcoholism and an extremely important risk factor for relapse to drinking in individuals attempting to maintain abstinence [1–3].

Studies on alcoholism have shown a contribution of both genetic and environmental components to its development. Specific genes that are associated with brain function and modulating neurotransmission may be important in assessing a person’s risk of developing alcoholism, possibly through their influence on certain personality features [4, 5].

The main neurotransmitter systems involved in developing alcohol dependence are gamma-aminobutyric acid (GABA), glutamate, dopamine, opioid, and serotonin systems. Disturbances in their metabolism and function are considered factors related to an increased risk of both alcohol and drug dependence, which may have a basis in abnormal brain function in the prefrontal cortex, the amygdala and the nucleus accumbens [1].

The role of vitamin D in the human body is more extensive than classically defined and goes beyond calcium homeostasis regulations, preventing rickets and osteomalacia. Vitamin D is involved in maintaining the proper function of the immune system, cardiovascular system, cellular proliferation, energy metabolism, and muscle strength [6]. Moreover, recent data suggest that 1,25(OH)_2_D_3_, a most-potent natural vitamin D metabolite, plays a role in the overall modulation of the central nervous system and may play a role in maintaining normal brain function [7, 8]. Vitamin D receptors (VDR) and activating enzymes (e.g., 1α-hydroxylase, the enzyme involved in the formation of the biologically active form of vitamin D) have been found in several areas of adult human brain, including the prefrontal cortex, cingulate gyrus, hippocampus, thalamus, hypothalamus, and substantia nigra [9]. The effect of vitamin D on the nervous system is associated with the regulation of the expression of genes involved with GABA-ergic neurotransmission and neuromediator synthesis [10–12]. In addition, vitamin D_3_ induces synthesis of neurotrophic factors such as nerve growth factor (NGF) and glial-cell-line-derived neurotrophic factor (GDNF) [13]. Moreover, vitamin D has been shown to be involved in neuroprotection and calcium homeostasis regulation by inducing the synthesis of Ca^2+^ binding proteins and modulating activity of the L-type calcium channels in neurons [14].

A few variants of the *VDR*, located on the long arm of chromosome 12 (12q13.1), have been found. Some of them are known to affect the VDR’s function, such as FokI (rs2228570, T/C) single nucleotide polymorphism (SNP). This SNP is located inside a start codon (ATG), and when the C variant is present, an alternative start site is used, leading to the expression of a shorter VDR protein (424 aa), which demonstrates a different activity than the longer one (427 aa) [15]. However, it has not yet been proven whether polymorphic variants of the *VDR* gene can affect the mental state of alcohol-dependent (AD) individuals by modulating expression of the array of genes related to central nervous system function. To date, *VDR* polymorphisms have been considered as potential contributing factors for developing schizophrenia as well as multiple sclerosis, but no significant relations were observed [16, 17]. In addition, vitamin D deficiency has been linked to an increased risk of developing major depression in both young adults [18] and elderly subjects [19].

Considering the function of vitamin D and the properties of the *VDR* FokI polymorphism we aimed the current study at assessing the association between *VDR* gene variants and impulsiveness in AD patients.

## Materials and methods

### Participants

The study group consisted of 148 AD patients, 106 males and 42 females. The participants were aged 23–71 years, with a mean age of 43.3 ± 9.8. They had been diagnosed as AD under the current DSM-IV definition [20] and were consequently admitted to residential addiction treatment programs at the Medical University of Warsaw. The median age of onset of drinking problems was 23 (IQR 19–31) years and the median duration of alcohol dependence was 15 (IQR 9–25) years. All patients were unrelated Caucasians, of Polish nationality, and were included in the study after written informed consent. The control group consisted of 212 subjects (112 unrelated healthy blood donors after relevant laboratory tests and consultations with appropriate physicians, and 100 healthy subjects attending a periodic general health checkup without history of alcoholism). The study was approved by the Medical University of Warsaw local bioethics committee and the Institutional Review Board at the University of Michigan.

### Measures

The Michigan Alcoholism Screening Test (MAST) was the instrument used to quantify severity of alcohol dependence. The MAST is a self-administered 25-item questionnaire, originally designed to identify probable cases of alcoholism [21]. Patients were also evaluated with the Mini International Neuropsychiatric Interview (M.I.N.I.), a short structured interview for both DSM-IV and ICD-10 psychiatric diagnoses [22].

The level of impulsivity was measured by the Barratt Impulsiveness Scale (BIS-11) [23, 24]. The BIS-11 is a subjective measure of impulsivity. It is a self-administered questionnaire, by which global impulsivity and its different dimensions are assessed. The six basic factors of impulsivity in BIS-11 are: motor impulsivity, attention factor, perseverance, cognitive instability, cognitive complexity and self-control. The three complex factors of impulsivity are combinations of basic factors: motor impulsivity (motor impulsivity as a basic factor and perseverance), non-planning impulsivity (self-control and cognitive complexity), and attentional impulsivity (attention factor and cognitive instability). In this study, the total scores of BIS-11 and scores for the three complex factors of impulsivity were analyzed.

### Genotyping

Peripheral blood samples were collected in EDTA tubes from both the patients and the controls. DNA was isolated according to the standard procedure and then stored at −20 °C until use.

The genotypes of the *VDR* FokI (rs2228570) SNP were analyzed in 148 patients and in 212 controls using a real-time PCR (polymerase chain reaction) method. Genotyping was carried out with the LightSNiP typing assay (TIB-MolBiol, Berlin, Germany) by analyzing the melting curves with the LightCycler^®^ 480 system available from Roche Diagnostics. Real-time PCR reactions were performed in 96-well PCR plates with cycling conditions as optimized by TIB-MolBiol.

### Statistical analyses

All genotyping results in AD patients and in controls were tested for Hardy–Weinberg Equilibrium (HWE) applying the HWSIM computer program (available at http://krunch.med.yale.edu/hwsim). All other tests were performed by the Statistica software package, version 9.0. Genotype and allele frequencies were compared between groups by Chi square statistics and Fisher’s exact test. The Kolmogorov–Smirnov test was used to test for normal distribution. Associations of clinical variables with genotypes of the candidate gene were examined with analysis of variance (ANOVA) and Student’s *t* test. The level of impulsivity was the primary outcome phenotype. Association tests were performed by means of ANOVA with the *VDR* FokI SNP (rs2228570; CC vs. CT vs. TT) as an independent variable and the BIS-11 scores entered as dependent variables. Tukey’s post hoc test was used for post hoc analysis when differences between groups were indicated by ANOVA. Continuous data are presented as means and standard deviations (SD). The criterion for significance was set at <0.05.

## Results

The genotype distribution for the *VDR* FokI polymorphism among AD subjects (χ^2^ = 2.79, df = 1, *P* = 0.09) and controls (χ^2^ = 3.02, df = 1, *P* = 0.08) was in Hardy–Weinberg equilibrium.

Comparison of AD patients and controls in respect to CC, CT and TT genotype distribution yielded no significant differences (*P* = 0.06). Frequencies of the C and T alleles were 0.56 and 0.44 both in patients and controls (Table 1). Also, no significant differences were observed in the comparison of CC and TT genotype frequencies between male and female AD patients (χ^2^ = 1.61, df = 1, *P* = 0.205; data not shown).

**Table 1:** Comparison of genotype and allele frequencies of *VDR* FokI polymorphism (rs2228570) in alcohol-dependent patients (AD) and control subjects

	*N*	Genotype frequencies			Allele frequencies	
		CC	CT	TT	C	T
AD patients	148	51 (34 %)	63 (43 %)	34 (23 %)	165 (0.56)	131 (0.44)
Controls	342	101 (29 %)	181 (53 %)	60 (18 %)	383 (0.56)	301 (0.44)
Statistics		χ^2^ = 4.62, df = 2, *P* = 0.099			χ^2^ = 0.01, df = 1, *P* = 0.94	

**Fig. 1:**
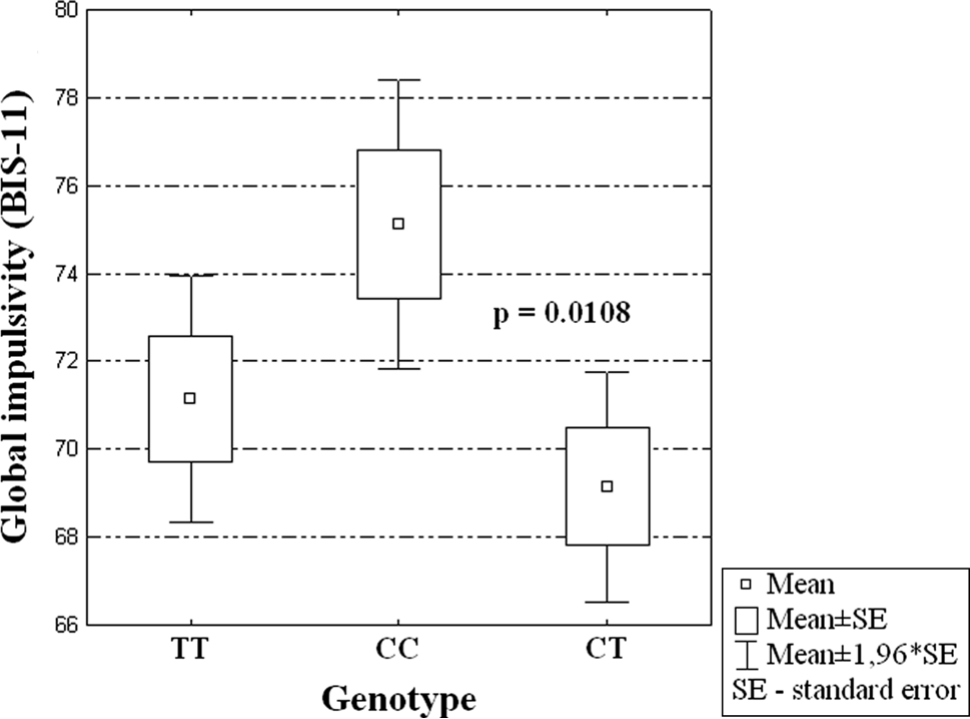
Association of CC genotype in FokI VDR SNP and level of global impulsivity in AD male patients (F = 4.424, df = 2, *P* = 0.014)

**Fig. 2:**
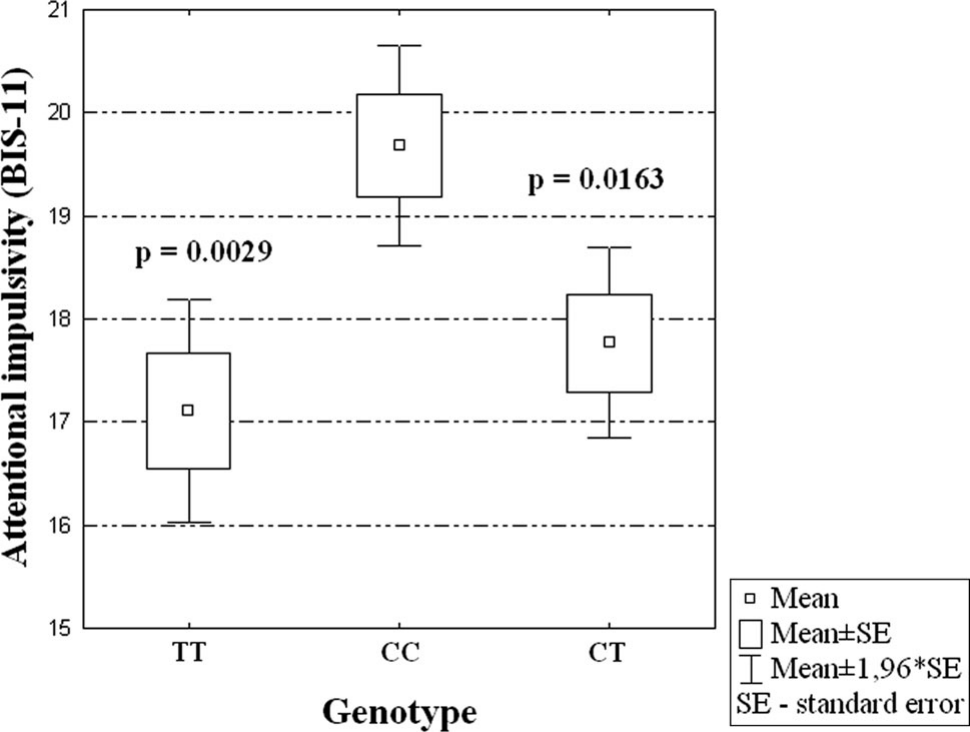
Association of CC genotype in FokI VDR SNP and level of attentional impulsivity in AD male patients (F = 6.600, df = 2, *P* = 0.002)

 In the group of male AD patients, the *VDR* FokI (rs2228570) polymorphism was associated with a significantly higher levels of both general (*P* = 0.014) and attentional impulsiveness (*P* = 0.002) based on BIS-11 scores (Table 2). After correction for multiple comparisons, in the subgroup of male patients it was found that subjects with CC genotype of the *VDR* gene had higher global impulsivity evaluated by the BIS-11 than patients with CT genotype (see Fig. 1). Moreover, male AD subjects with CC genotype had higher attentional impulsiveness scores than the patients with the other two genotypes (TC or TT) (see Fig. 2).

**Table 2:** Relationships between different types of impulsivity and *VDR* gene FokI polymorphism in male alcohol-dependent patients

FokI *VDR* polymorphism	Global impulsivity (BIS-11)	Attentional impulsivity (BIS-11)	Motor impulsivity (BIS-11)	Nonplanning impulsivity (BIS-11)
CC (*n* = 35)	75.11 ± 9.92	19.69 ± 2.94	26.06 ± 4.72	29.37 ± 4.58
CT (*n* = 43)	69.14 ± 8.77	17.77 ± 3.08	23.98 ± 3.49	27.39 ± 4.59
TT (*n* = 28)	71.14 ± 7.59	17.11 ± 2.94	24.64 ± 3.78	29.39 ± 2.85
ANOVA	F = 4.424, df = 2 *P* = 0.014	F = 6.600, df = 2 *P* = 0.002	F = 2.647, df = 2 *P* = 0.076	F = 2.850, df = 2 *P* = 0.062

The values presented are arithmetic means and standard deviations (mean ± SD)

*BIS-11* Barratt’s impulsiveness scale, *ANOVA* analysis of variance

In the female AD subjects, no significant relationship between the genotype distribution and total (F = 0.402, df = 2, *P* = 0.67) or attentional impulsiveness (BIS-11; F = 0.450, df = 2; *P* = 0.64) were found (data not shown).

## Discussion

The main finding of this study is that genetic variance in the *VDR* gene is related to differences in impulsivity of male AD patients. It has been found that the *VDR* FokI (rs2228570) polymorphism is associated to a significant degree with the general and attentional impulsiveness of the AD males. In both cases, individuals with the CC genotype had the highest impulsivity scores.

On the other hand, other studies have reported that, in a rat model of vitamin D deficiency offspring showed severe deficits in brain development and function [11, 25]. In addition, vitamin D deficiency-induced behavioral changes in prenatally depleted animals (DVD-deficient rats) include impaired attention on a latent inhibition task, altered learning and hyperlocomotion compared to control animals [26–28].

In AD patients, low plasma concentrations of vitamin D may be expected as reported by González-Reimers et al. [29, 30]. A low vitamin D level, along with the occurrence of particular variants of the *VDR* gene resulting in differences in VDR receptor structure and activity, may jointly determine levels of impulsivity and in the present study may account for the higher levels of both global and attentional impulsivity in male AD patients with CC FokI genotype.

Although our study suggests an association between FokI *VDR* gene polymorphism and levels of impulsivity in male alcoholic individuals, the results need to be replicated on a larger sample. Alcohol is a toxic compound leading to neurodegeneration. A better knowledge of genetic determinants and more accurate concept of what effects a vitamin D deficiency has are fundamental for understanding the homeostatic mechanisms in the central nervous system; and perhaps, in the future, this concept may make it possible to assess the processes that prevent brain damage in AD patients.

In a study by Saunders et al. [31] it was shown that patients who themselves were not alcoholics but had AD family members displayed greater impulsive behavior than those without such a familial background. This relationship applies primarily to men, which is consistent with the differences in heritability of the disorder between males and females as reported in the literature. In the present study, there was no effect of the FokI genotype on impulsivity levels in alcoholic women. Hence, there may exist a gender-dependent effect, which has been also suggested to the relationship between *VDR* gene polymorphism and development of diabetes type I [32]. The reasons for these differences can probably result from the particular effects of the gender-specific hormones (particularly estrogen in women) on gene expression. Estrogens influence the extent of the *VDR* gene expression [33], and use of estrogens by women leads to increased expression of the genes of the receptors for active form of vitamin D [34]. However, small number of women in the present study limits the conclusions that can be drawn; our results can only suggest that genetic background might have a stronger influence on impulsivity on men than on women. Therefore, further studies are needed to make a final conclusion.

An additional limitation of the present study is that plasma vitamin D concentration was not measured, which could have added stronger support to the final conclusions and could have been correlated with the *VDR* genotype-dependent differences in activity and its influence on the level of impulsivity. However, it is well known that genetics—including genetic polymorphism—determines the diversity between individuals and thus also each person’s temperament, especially impulsivity. Neurotransmission is based on Ca^2+^ flow, which regulation involves vitamin D. The capability of this regulation may depend on molecular differences in the receptor’s structure.

To the best of our knowledge, the present study is the first to report an association between a polymorphism in the *VDR* gene and a psychometrically-derived impulsiveness trait. Our data suggest that common variants in *VDR* contribute to high levels of impulsivity, but await verification by independent research. Replication of our analysis in other studies would strengthen the putative link between FokI polymorphism and impulsivity, and would establish *VDR* as a CNS modulator gene. In conclusion, we are reporting for the first time that genetic variation in *VDR* is associated with impulsivity in male AD subjects.
